# Supplementary material for: SHOC2 phosphatase-dependent RAF dimerization mediates resistance to MEK inhibition in RAS-mutant cancers
Source: Nat Commun. 2019 Jun 10;10:2532. doi: 10.1038/s41467-019-10367-x (PMC6557854; doi:10.1038/s41467-019-10367-x)
Supplement: Supplementary file 1 — Supplementary Information [file 41467_2019_10367_MOESM1_ESM.pdf]

Supplementary Information

Title

SHOC2 phosphatase-dependent RAF dimerization mediates resistance to MEK inhibition in RAS-mutant cancers

Authors

Greg G Jones[1], Isabel Boned del Rio[1], Sibel Sari[1], Aysen Sekerim[1], Lucy C Young[1], Nicole Hartig[1], Itziar Areso Zubiaur[1], Mona A.El-Bahrawy[3], Rob E Hynds[1], Winnie Lei[1], Miriam Molina-Arcas[2], Julian Downward[2], Pablo Rodriguez-Viciana[1,*].

Author Affiliations

[1]University College London Cancer Institute, London WC1E 6DD, UK

[2]The Francis Crick Institute, 1 Midland Road, London NW1 1AT, UK; Lung Cancer Group, Division of Molecular Pathology, The Institute of Cancer Research, 237 Fulham Road, London SW3 6JB, UK

[3]Department of Histopathology, Imperial College London, Du Cane Road, London W12 0NN, UK

[*]Correspondence: p.rodriguez-viciana@ucl.ac.uk

Cell Line	Cancer Subtype	KRAS	NRAS	EGFR	BRAF	STK11	TRP53
**A549**	NSCLC	**G12S**				Q37*	
**H460**	NSCLC	**Q61H**				Q37*	
**A427**	NSCLC	**G12D**				Ex.1-5 Deletion	
**H23**	NSCLC	**G12C**				W322*	M246I
**H358**	NSCLC	**G12C**					
**H1792**	NSCLC	**G12C**					
**H2009**	NSCLC	**G12A**					R273L
**H727**	NSCLC	**G12V**			ARAF A285D		Q165_S166insYKQ
**H1944**	NSCLC	**G13D**				K78N K62N	
**PATU-8092**	Pancreatic	**G12V**					C176S
**HCT116**	Colorectal	**G13D**					
**MDA-MB-231**	TNBC	**G13D**			G464V		R280K
**SK-MEL-2**	Melanoma		**Q61R**				G245S
**PC9 (PC-14)**	NSCLC			**E746_A750del**			R248Q
**PC9-er**	NSCLC			**E746_A750del T790M**			R248Q
**HCC827**	NSCLC			**E746_A750del**			V218delV
**HCC4006**	NSCLC			**E746_A750del**			
**CAL-12T**	NSCLC				**G466V**		C135F
**NCI-H1395**	NSCLC				**G469V**		
**HT-29**	Colorectal				**V600E**		R273H
**H522**	NSCLC						P191fs*56
**H226**	NSCLC						
**H520**	NSCLC						W146*
**NL20**	Non-cancer Lung epithelial cells						

Driver mutation in indicated by bold text. Mutational status supplied by https://cansarblack.icr.ac.uk/

Supplementary Table 1 | Cell Lines and associated driver mutations used in this research

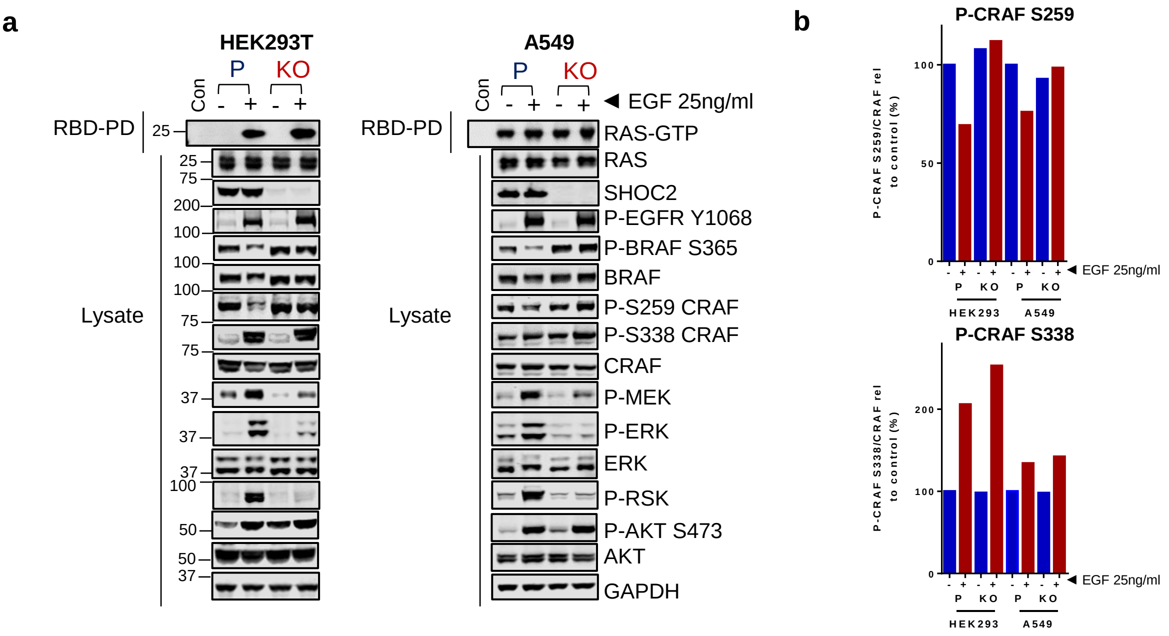

Supplementary Figure 1 | SHOC2 is required for S365/S259 B/CRAF dephosphorylation and downstream ERK-pathway activation by EGF but not RAS activation or CRAF S338 phosphorylation. Related to Figure 2.

- **a)** Lysates were harvested from indicated cells treated with 25ng/ml of EGF for 10minutes and used to perform RAS-RBD pull downs or to probe by western blot with indicated antibodies. In RAS wt HEK293T cells, EGF potently stimulates RAS activation and S338 CRAF phosphorylation, and SHOC2 downregulation does not have any significant effect. In RAS mutant A549 cells, basal RAS-GTP levels and S338 CRAF phosphorylation are high and EGF does not stimulate any detectable increase.
- **b)** Lysates from (a) are quantified for P-CRAF S259 and S338/ total CRAF.

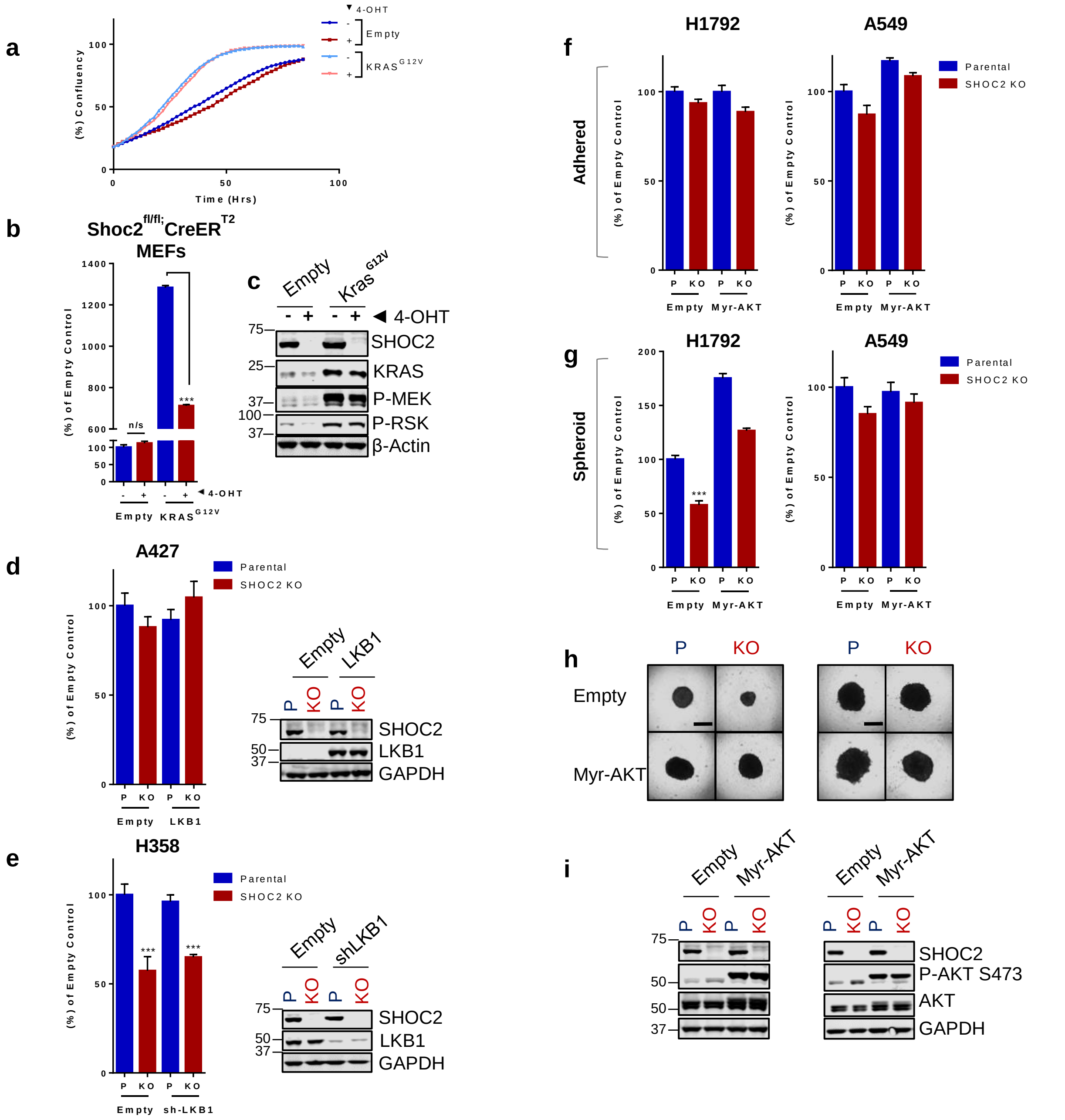

Supplementary Figure 2 | AKT signalling can overcome the SHOC2 requirement for 3D growth in RAS-mutant cells. Related to Figure 2.

- **a)** SHOC2 is not required for adhered cell growth of control or KRAS^{G12V} expressing MEFs. E6-immortalized MEFs derived from Shoc2$^{fl/fl}$;CreER^{T2} mice and infected with retrovirus expressing KRAS^{G12V} or an empty vector control were seeded at low density and proliferation rate monitored by incucyte growth curves.
- **b)** SHOC2 is required for 3D growth in KRAS^{G12V} expressing MEFs. Cells used in A were seeded in low-attachment plates and growth determined at Day 5 by alamar blue staining (mean ± SD) (n=3). Significance is determined using a two tailed T-test $*p < 0.05$, $**p < 0.01$ or $***p < 0.001$.
- **c)** Lysates of cells described in (**a-b**) were probed with indicated antibodies.

d) Parental or SHOC2 KO A427 cells with stable re-experession of LKB1 (or empty vector control) were seeded either under anchorage-independent conditions and growth determined as (**b**) or for lysates probed with indicated antibodies.

e) Parental or SHOC2 KO H358 cells infected with control or shLKB1 viruses were either seeded under anchorage-independent conditions and growth determined as (**b**) or for lysate and lysates probed with indicated antibodies.

f) Expression MYR-AKT has no effect on anchorage-dependent growth in H1792 or A549 cells. Growth was determined at Day 5 by alamar blue staining (mean ± SD) (n=4). Significance is determined using a two tailed T-test ∗$p < 0.05$, ∗∗$p < 0.01$ or ∗∗∗$p < 0.001$.

g) Inhibition of anchorage independent growth in SHOC2 K0 H1792 cells is rescued by MYR-AKT expression. Cells described in (**f**) were seeded under anchorage-independent conditions and growth determined as (**b**).

h) P/C images of representative spheroids measured in (**G**) at D5. Scale bar = 200µm.

i) Lysates of cells from (**f-h**) were probed with indicated antibodies.

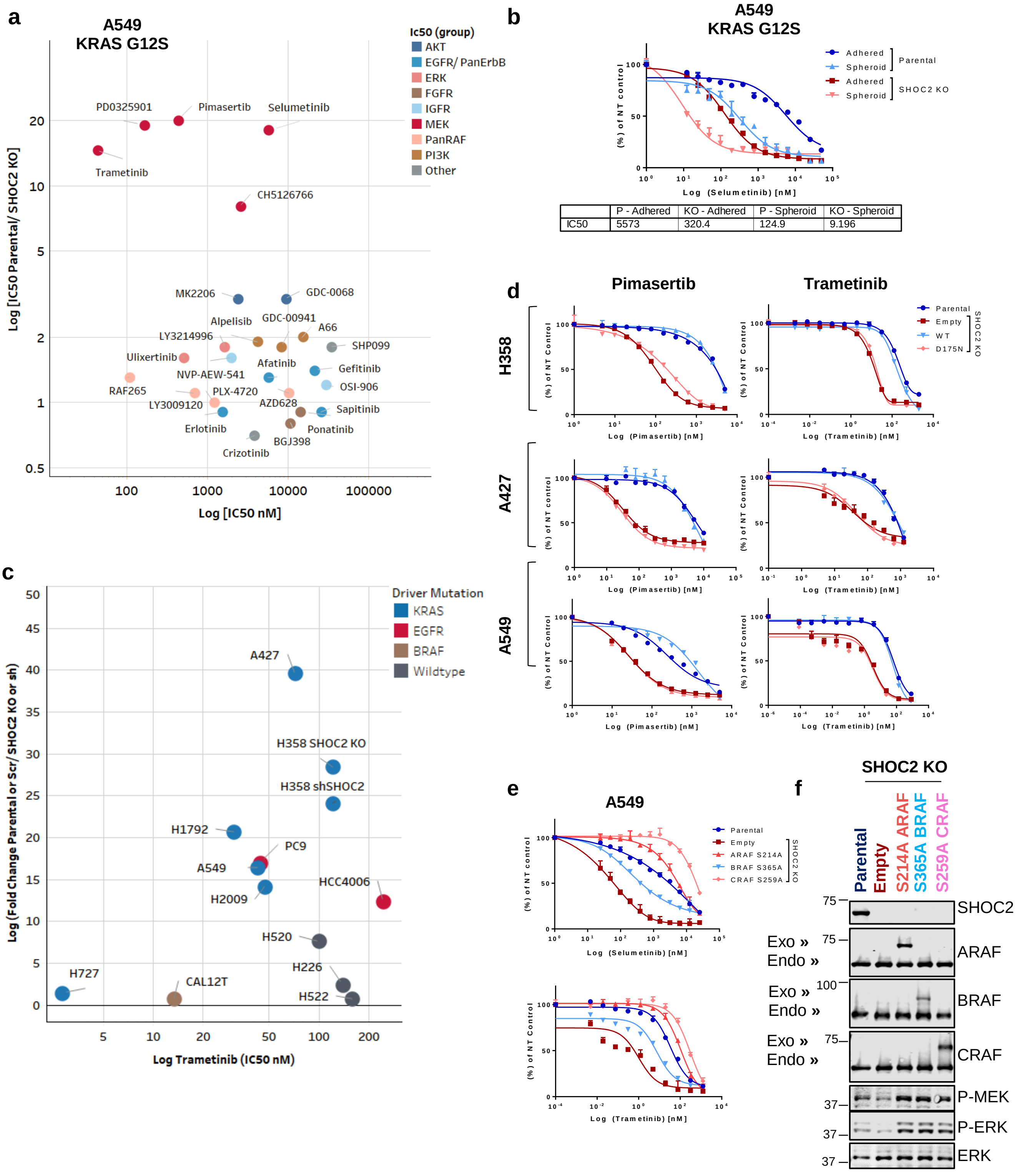

	P - Adhered	KO - Adhered	P - Spheroid	KO - Spheroid
IC50	5573	320.4	124.9	9.196

Supplementary Figure 3 | SHOC2 deletion sensitizes RAS- and EGFR-mutant NSCLC cells to MEKi's. Related to Figure 3.

- **a)** Viability assays for Parental or SHOC2 KO A549 cells treated with the indicated inhibitors. The resulting IC50 values are plotted (x-axis) and compared against the fold change between the IC50 value determined for the Parental and SHOC2 KO cells (y-axis).
- **b)** SHOC2 depletion similarly sensitises A549 cells to MEKi's in adhered or spheroid culture conditions. Viability assays for Parental or SHOC2 KO A549 cells treated with the indicated inhibitors in adhered or spheroid culture conditions.
- **c)** SHOC2 deletion sensitises KRAS- and EGFR-mutant, but not BRAF-mutant or wildtype NSCLC cell lines to the MEKi Trametinib. Fold change in IC50 value between shSCR and shSHOC2 or parental and SHOC2 KO cells plotted for each cell line.
- **d)** Sensitisation of SHOC2 KO NSCLC cell lines to MEKi's is rescued by re-expression of WT- but not D175N-SHOC2. Viability assays were performed for SHOC2 KO cells after stable expression of WT-SHOC2, SHOC2 D175N or empty vector control.
- **e)** Sensitisation of SHOC2 knockout NSCLC cell lines to MEKi's is rescued by expression of RAF 'S259' phosphorylation-deficient mutants. Viability assays were performed for A549 SHOC2 KO cells after stable expression of S214A ARAF, S365A BRAF, S259A CRAF or empty vector control.

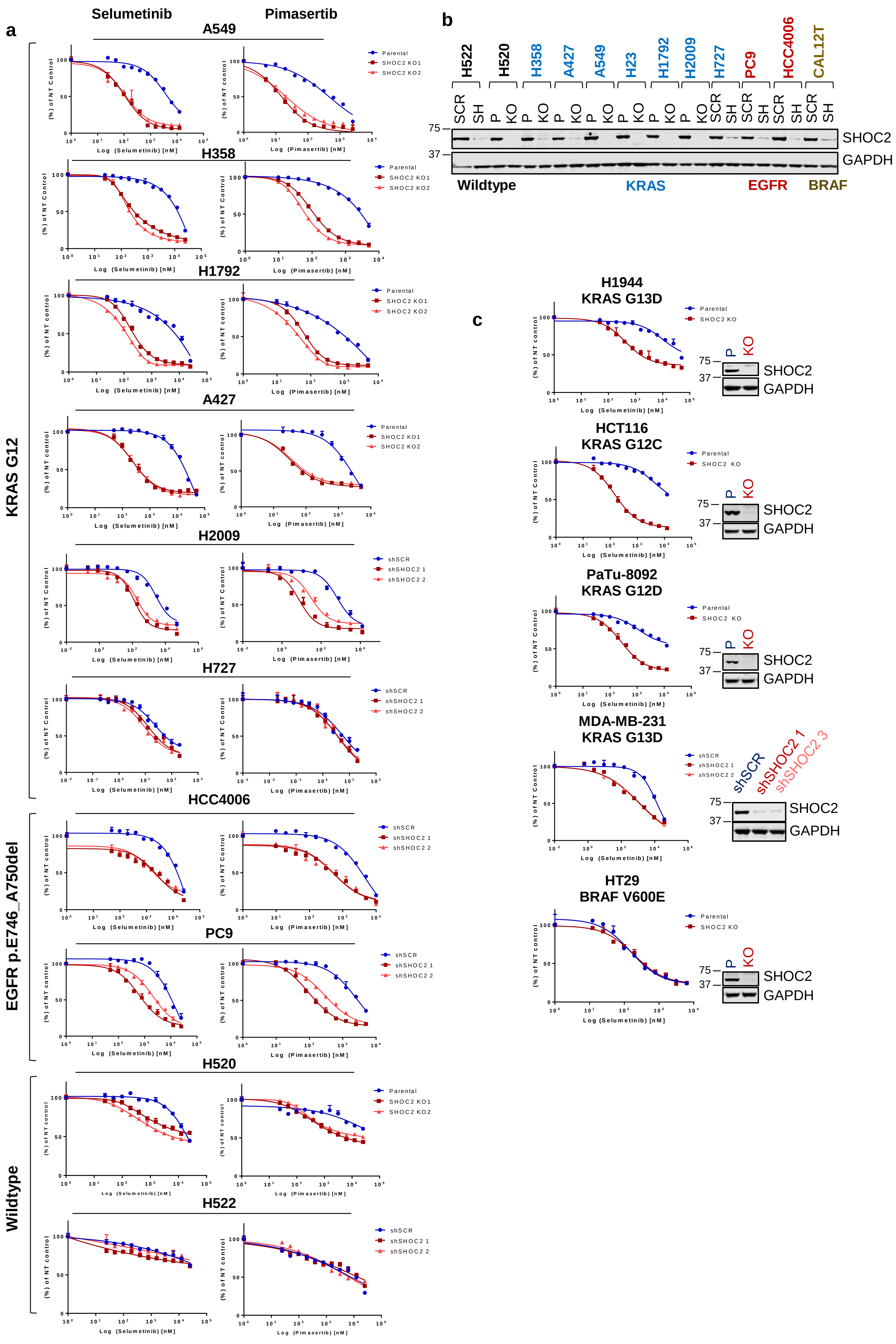
a
Selumetinib
Pimasertib
A549
H358
H1792
A427
H2009
H727
HCC4006
PC9
H520
H522
KRAS G12
EGFR p.E746_A750del
Wildtype
Parental
SHOC2 KO1
SHOC2 KO2
shSCR
shSHOC2 1
shSHOC2 2
(%) of NT Control
Log (Selumetinib) [nM]
Log (Pimasertib) [nM]
b
H522
H520
H358
A427
A549
H23
H1792
H2009
H727
PC9
HCC4006
CAL12T
SCR
SH
P
KO
75
37
SHOC2
GAPDH
Wildtype
KRAS
EGFR
BRAF
c
H1944
KRAS G13D
HCT116
KRAS G12C
PaTu-8092
KRAS G12D
MDA-MB-231
KRAS G13D
HT29
BRAF V600E
Parental
SHOC2 KO
shSCR
shSHOC2 1
shSHOC2 2
shSHOC2 3
P
KO
SHOC2
GAPDH
(%) of NT control
Log (Selumetinib) [nM]

Supplementary Figure 4 | Characterisation of SHOC2 KD/ KO cell lines
Related to Figure 3

a) SHOC2 KD/ KO sensitises RAS- and EGFR-mutant but not wildtype cell lines to MEKi's. Representative viability assays are shown for the indicated NSCLC cell lines treated with either Selumetinib or Pimasertib. SHOC2 was inhibited by shRNA knockdown (KD) or CRISPR-mediated knockout (KO) as indicated.

b) Lysates of cells described in (**a**) were probed with the indicated antibodies.

c) SHOC2 KD/ KO sensitises RAS- but not BRAF V600E-mutant cell lines from different tissue types to MEKi's. Representative viability assays are shown for the indicated cell lines treated with Selumetinib. SHOC2 was inhibited by shRNA knockdown (KD) or Lenti-CRISPR-mediated knockout (KO) as indicated.

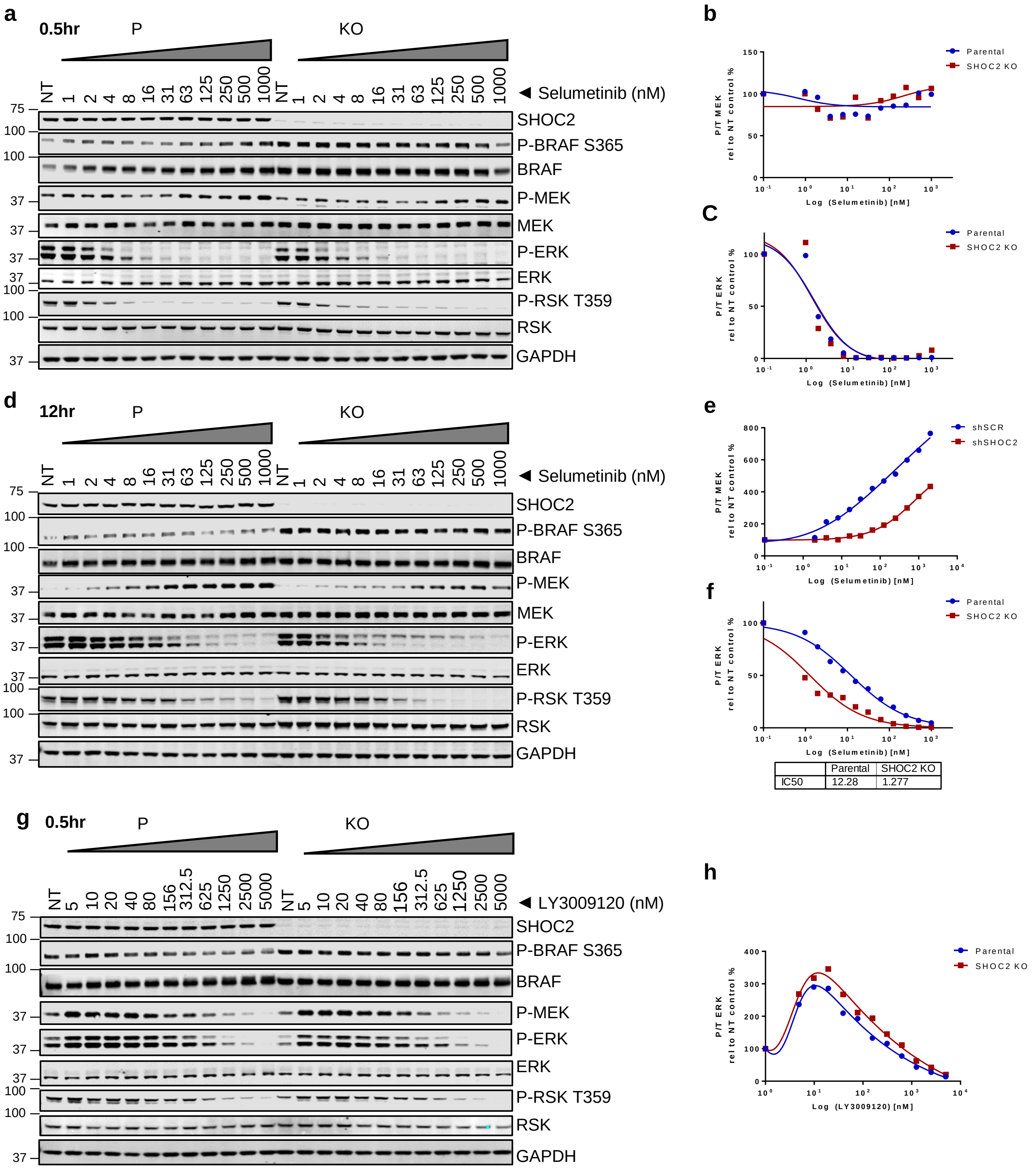

Supplementary Figure 5 | SHOC2 deletion impairs rebound MEK phosphorylation by Selumetinib in H358 cells in both a dose and time-dependent manner. Related to Figure 4.

- **a)** Cells were treated for 0.5hr with the indicated doses of Selumetinib and lysates probed with indicated antibodies.
- **b)** Quantification of P-MEK/ T-MEK in (**a**) relative to NT.
- **c)** Quantification of P-ERK/ T-ERK in (**a**) relative to NT.
- **d)** Cells were treated for 12hr with the indicated doses of Selumetinib and lysates probed with indicated antibodies.
- **e)** Quantification of P-MEK/ T-MEK in (**d**) relative to P-MEK NT.
- **f)** Quantification of P-ERK/ T-ERK in (**d**) relative to P-ERK NT.

g) Cells were treated for 0.5hr with the indicated doses of LY3009120 and lysates probed with indicated antibodies

h) Quantification of P-ERK/ T-ERK in (**g**) relative to P-ERK NT.

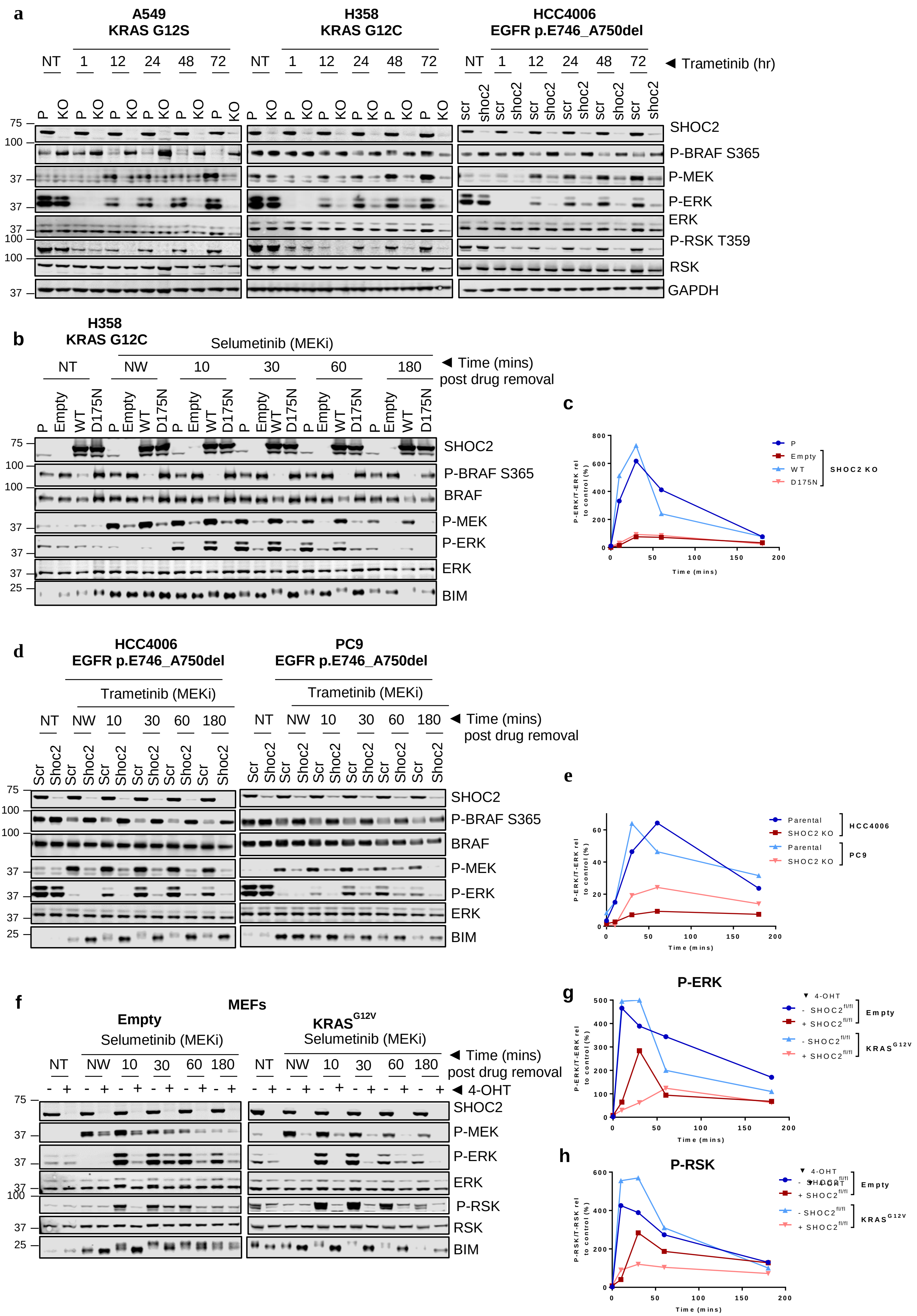
a
A549
KRAS G12S
H358
KRAS G12C
HCC4006
EGFR p.E746_A750del
NT 1 12 24 48 72
◀ Trametinib (hr)
P KO
scr shoc2
SHOC2
P-BRAF S365
P-MEK
P-ERK
ERK
P-RSK T359
RSK
GAPDH
75
100
37
b
H358
KRAS G12C
Selumetinib (MEKi)
NT NW 10 30 60 180
◀ Time (mins)
post drug removal
P Empty WT D175N
SHOC2
P-BRAF S365
BRAF
P-MEK
P-ERK
ERK
BIM
25
c
P
Empty
WT
D175N
SHOC2 KO
P-ERK/T-ERK rel to control (%)
Time (mins)
d
HCC4006
EGFR p.E746_A750del
PC9
EGFR p.E746_A750del
Trametinib (MEKi)
Scr Shoc2
e
Parental
SHOC2 KO
HCC4006
PC9
f
MEFs
Empty
KRAS G12V
Selumetinib (MEKi)
◀ 4-OHT
P-RSK
g
P-ERK
4-OHT
- SHOC2 fl/fl
+ SHOC2 fl/fl
Empty
KRAS G12V
h
P-RSK
P-RSK/T-RSK rel to control (%)
Time (mins)

Supplementary Figure 6 | SHOC2 deletion impairs rebound MEK phosphorylation by Selumetinib in H358 cells in both a dose and time-dependent manner. Related to Figure 4.

- **a)** Cells were treated for 0.5hr with the indicated doses of Selumetinib and lysates probed with indicated antibodies.
- **b)** Quantification of P-MEK/ T-MEK in (**a**) relative to NT.
- **c)** Quantification of P-ERK/ T-ERK in (**a**) relative to NT.
- **d)** Cells were treated for 12hr with the indicated doses of Selumetinib and lysates probed with indicated antibodies.
- **e)** Quantification of P-MEK/ T-MEK in (**d**) relative to P-MEK NT.
- **f)** Quantification of P-ERK/ T-ERK in (**d**) relative to P-ERK NT.
- **g)** Cells were treated for 0.5hr with the indicated doses of LY3009120 and lysates probed with indicated antibodies
- **h)** Quantification of P-ERK/ T-ERK in (**g**) relative to P-ERK NT.

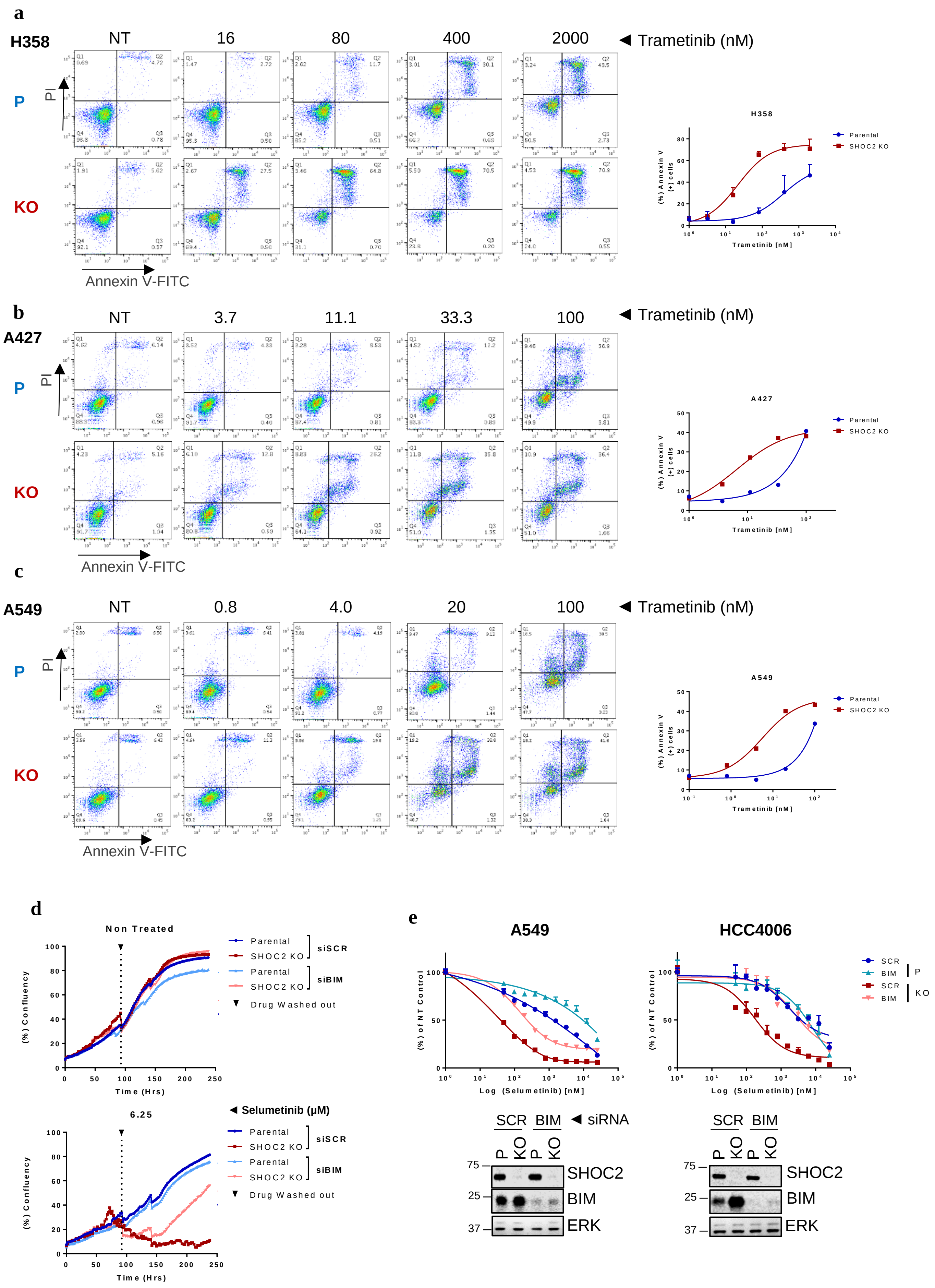

a
H358
NT
16
80
400
2000
◀ Trametinib (nM)
P
KO
PI
Annexin V-FITC
H358
Parental
SHOC2 KO
(%) Annexin V (+) cells
Trametinib [nM]
b
A427
NT
3.7
11.1
33.3
100
◀ Trametinib (nM)
P
KO
PI
Annexin V-FITC
A427
Parental
SHOC2 KO
(%) Annexin V (+) cells
Trametinib [nM]
c
A549
NT
0.8
4.0
20
100
◀ Trametinib (nM)
P
KO
PI
Annexin V-FITC
A549
Parental
SHOC2 KO
(%) Annexin V (+) cells
Trametinib [nM]
d
Non Treated
Parental
SHOC2 KO
siSCR
Parental
SHOC2 KO
siBIM
Drug Washed out
(%) Confluency
Time (Hrs)
◀ Selumetinib (μM)
6.25
Parental
SHOC2 KO
siSCR
Parental
SHOC2 KO
siBIM
Drug Washed out
(%) Confluency
Time (Hrs)
e
A549
HCC4006
SCR
BIM
P
SCR
BIM
KO
(%) of NT Control
Log (Selumetinib) [nM]
SCR
BIM
◀ siRNA
P
KO
P
KO
75
25
37
SHOC2
BIM
ERK
SCR
BIM
P
KO
P
KO
75
25
37
SHOC2
BIM
ERK

Supplementary Figure 7 | Combined genetic inhibition of SHOC2 and MEKi treatment promote BIM-dependent apoptosis in RAS- and EGFR mutant cells. Related to Figure 6.

- **a)** Parental and SHOC2 KO H358 cells were treated with the indicated doses of Trametinib for 48hr and analysed by FACS after staining with PI and Annexin V. Representative profiles are displayed from n=3 experiments and (%) Annexin V positive cells quantified from n=3 experiments.
- **b)** As (**a**) for A427 cells.
- **c)** As (**a**) for A549 cells.
- **d)** Incucyte growth curves of Parental or SHOC2 KO H358 cells transfected with SCR or BIM siRNAs were grown in the presence of single addition of indicated concentrations of Selumetinib. After 96 hr the inhibitor was washed out and cell growth measured for an additional 4-days by incucyte imaging.
- **e)** BIM knockdown diminishes the sensitisation of RAS- and EGFR-mutant cells to combined SHOC2 depletion and MEK inhibition. A549 P and SHOC2 KO or HCC4006 shSCR and shSHOC2 cells transfected with SCR or BIM siRNAs were treated with Selumetinib on Day 2 post transfection and viability assays performed after 96hr.

A B C

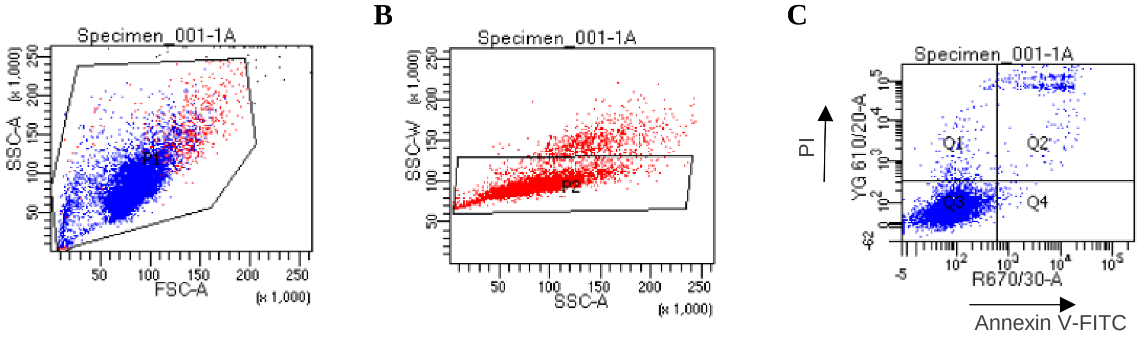

D

Tube: 1A

Population	#Events	%Parent	%Total
All Events	10,000	####	100.0
P1	9,929	99.3	99.3
P2	9,140	91.4	91.4
Q1	174	1.9	1.7
Q2	320	3.5	3.2
Q3	8,609	94.2	86.1
Q4	37	0.4	0.4

A- All events gated
B- Single cells gated
C- PI vs ANNEXIN V staining separates Q1 – Debris, Q2 – Late Apoptosis, Q3 – Live cells, Q4- Early Apoptosis.
D- (%) of gated cells compared to total cell input is shown for A-C.

Supplementary Figure 8 | FACS gating strategy

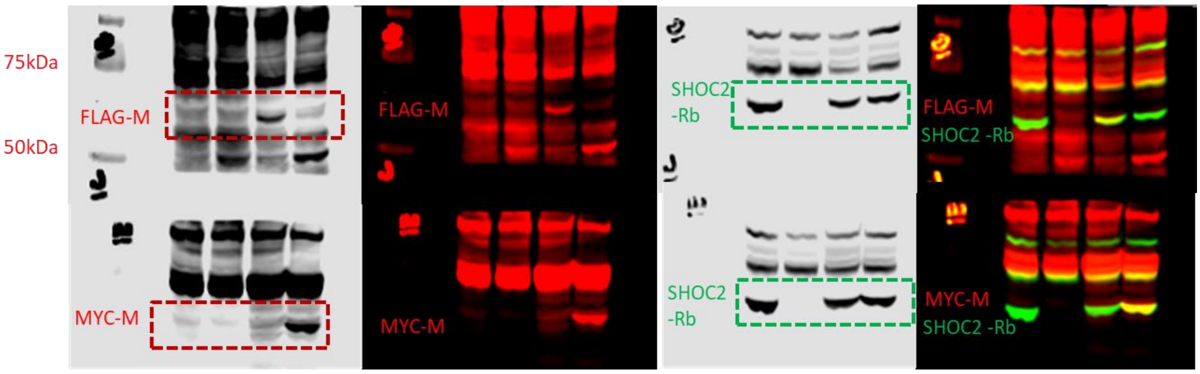

Supplementary Figure 9 | Uncropped western blots of the most important blots Fig. 1b

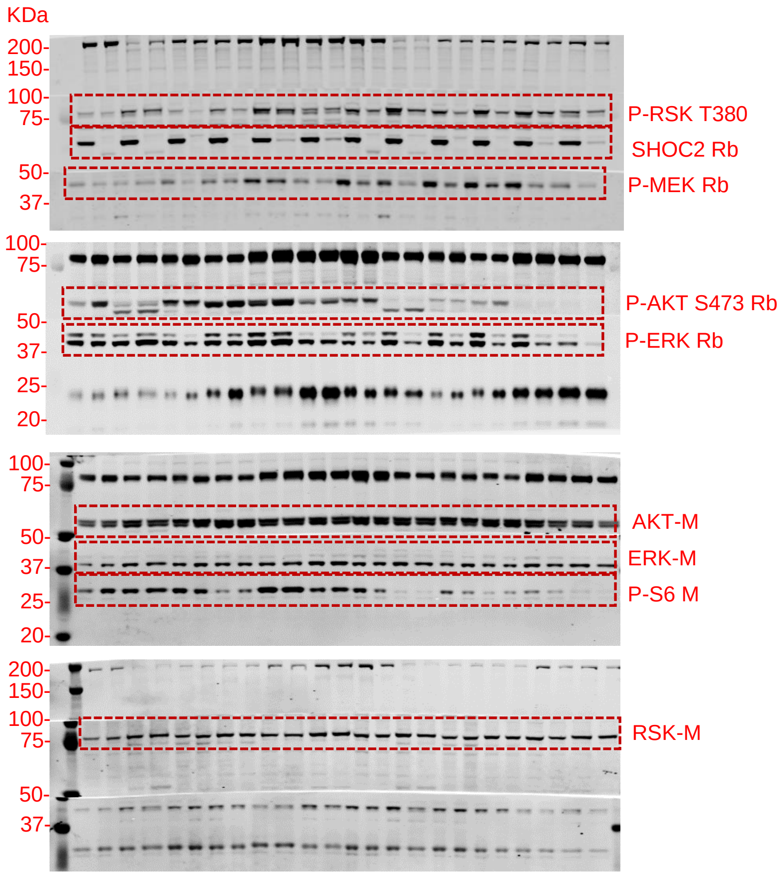

Supplementary Figure 10 | Uncropped western blots of the most important blots Fig. 2i

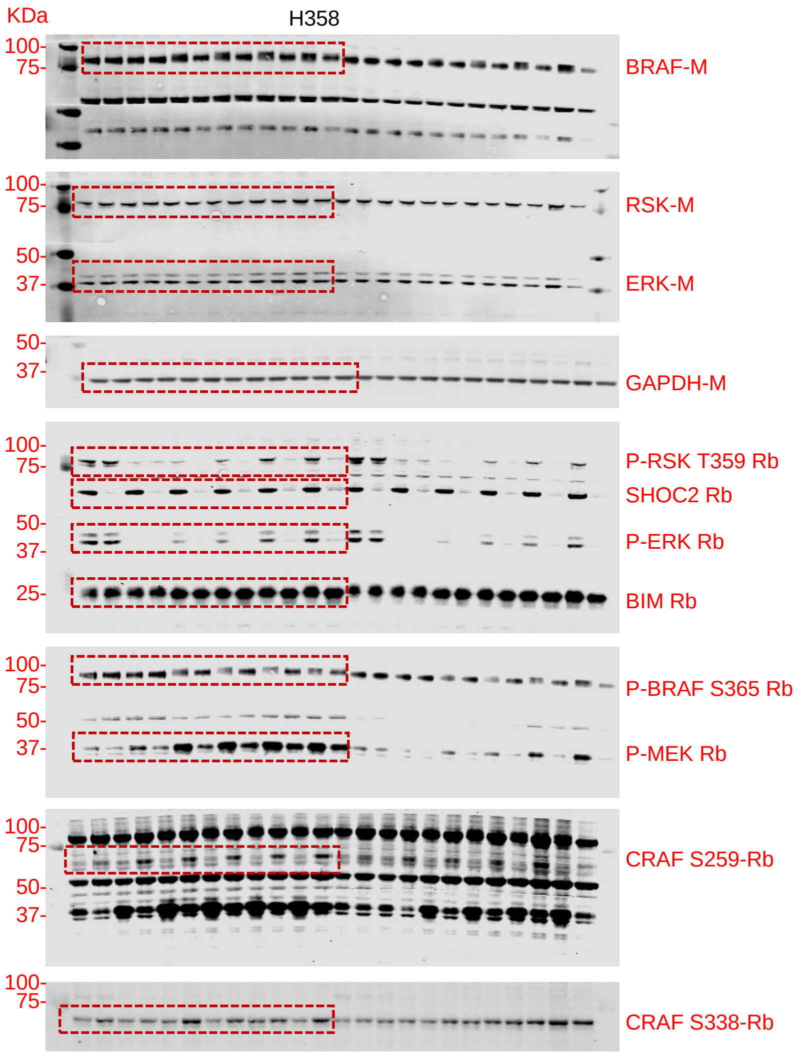

KDa
H358
100-
75-
BRAF-M
100-
75-
RSK-M
50-
37-
ERK-M
50-
37-
GAPDH-M
100-
75-
P-RSK T359 Rb
SHOC2 Rb
50-
37-
P-ERK Rb
25-
BIM Rb
100-
75-
P-BRAF S365 Rb
50-
37-
P-MEK Rb
100-
75-
50-
37-
CRAF S259-Rb
100-
75-
CRAF S338-Rb

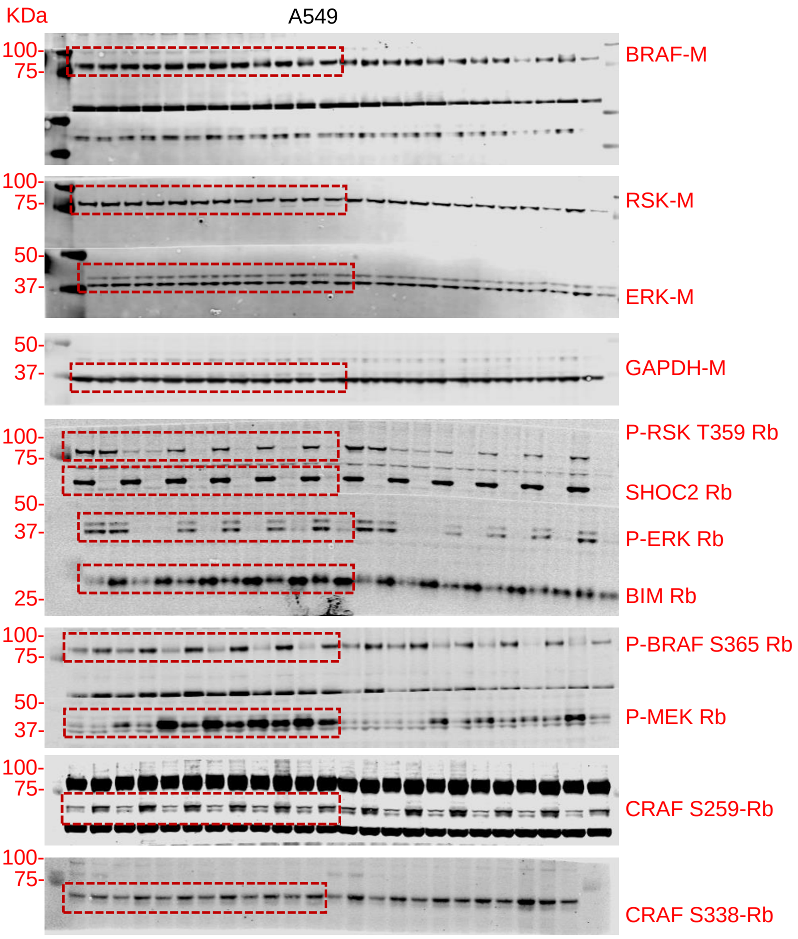
KDa
A549
100-
75-
BRAF-M
100-
75-
RSK-M
50-
37-
ERK-M
50-
37-
GAPDH-M
100-
75-
P-RSK T359 Rb
SHOC2 Rb
50-
37-
P-ERK Rb
25-
BIM Rb
100-
75-
P-BRAF S365 Rb
50-
37-
P-MEK Rb
100-
75-
CRAF S259-Rb
100-
75-
CRAF S338-Rb

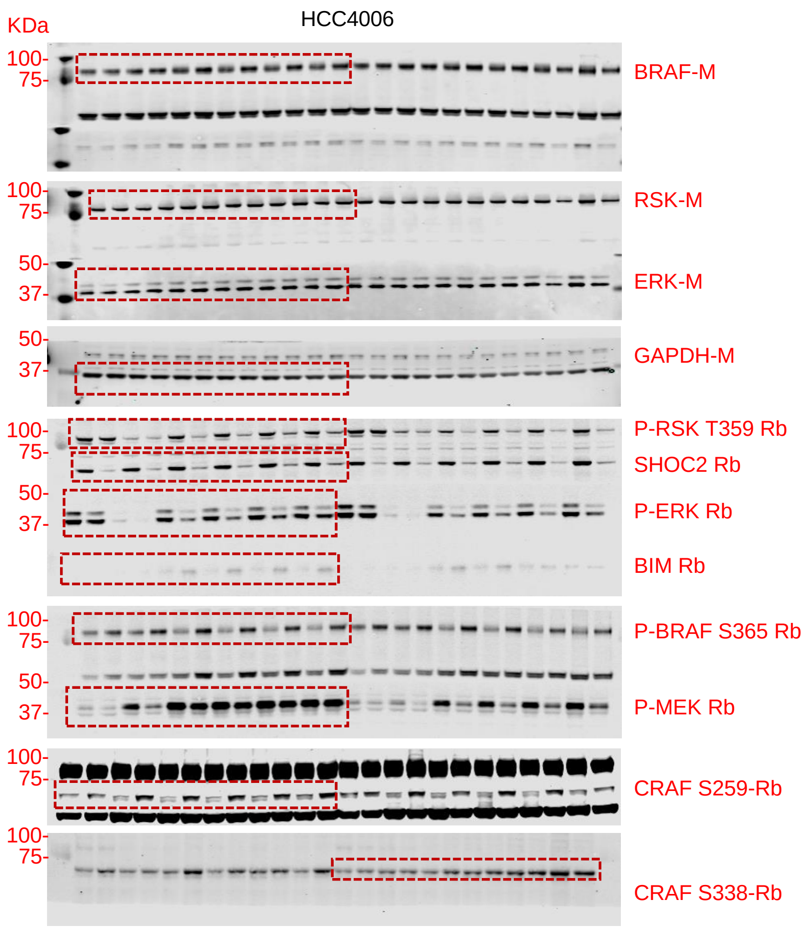

Supplementary Figure 11 | Uncropped western blots of the most important blots Fig. 4a

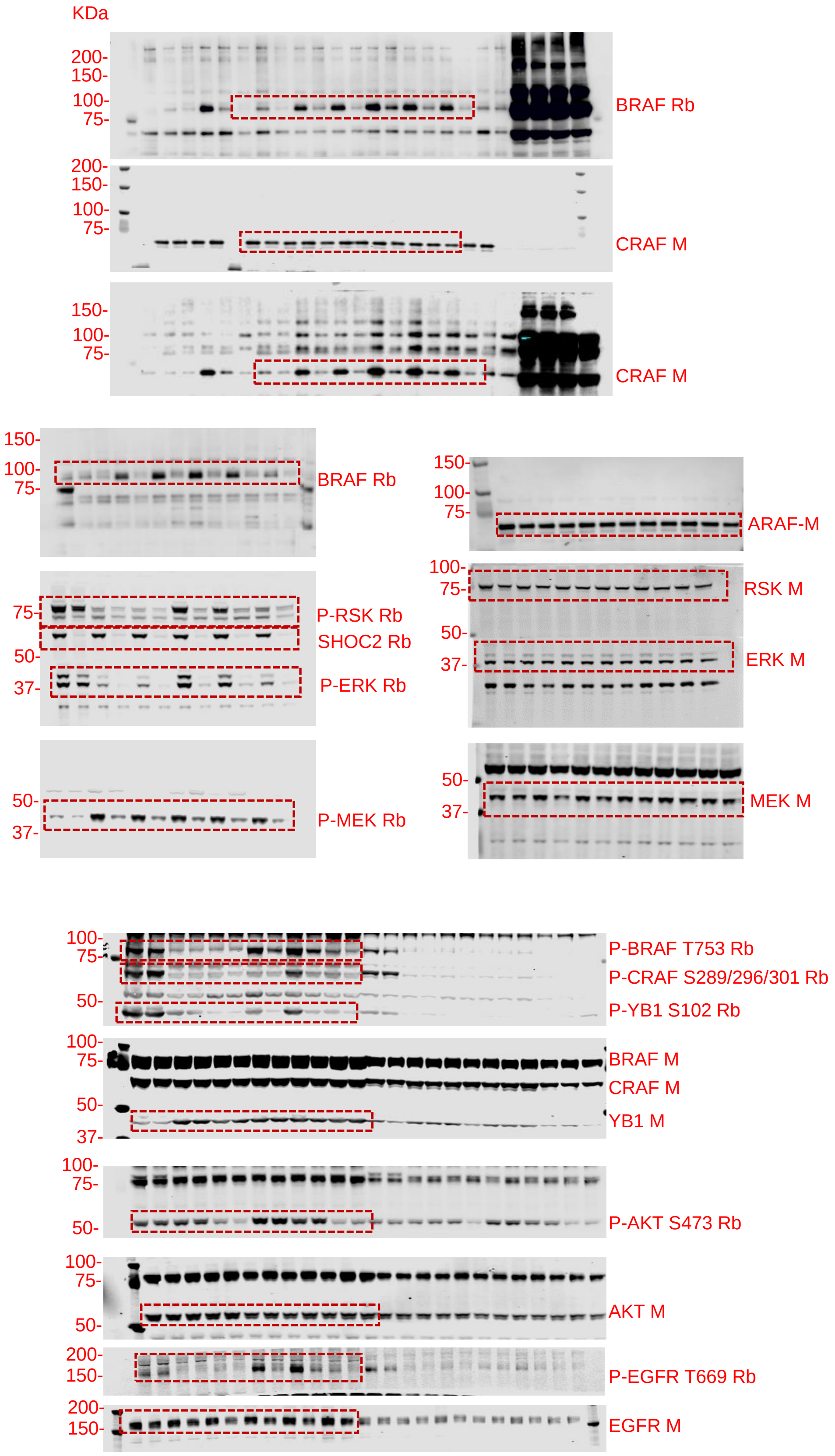

Supplementary Figure 12 | Uncropped western blots of the most important blots Fig. 5a

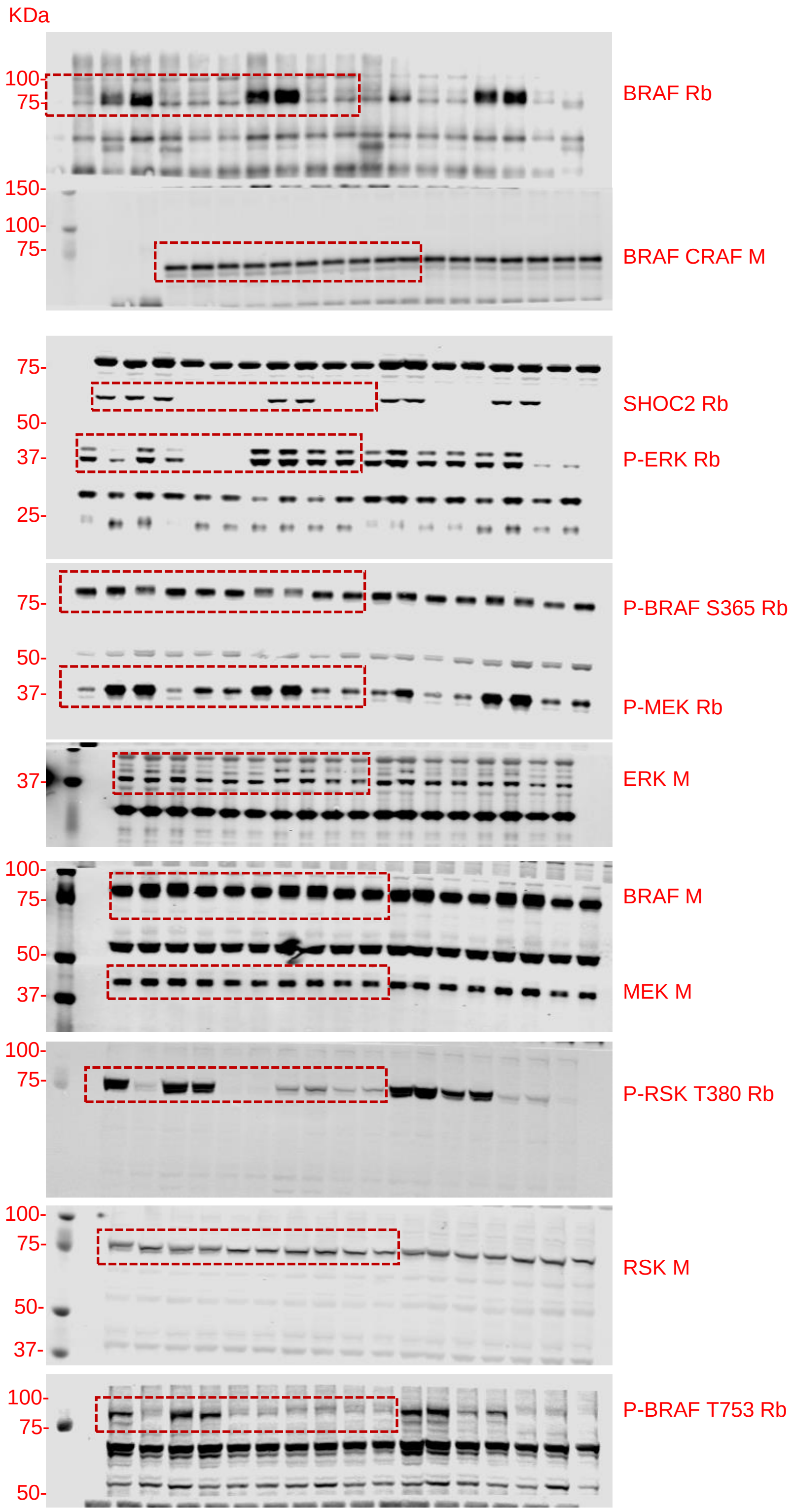

Supplementary Figure 13 | Uncropped western blots of the most important blots Fig. 5d

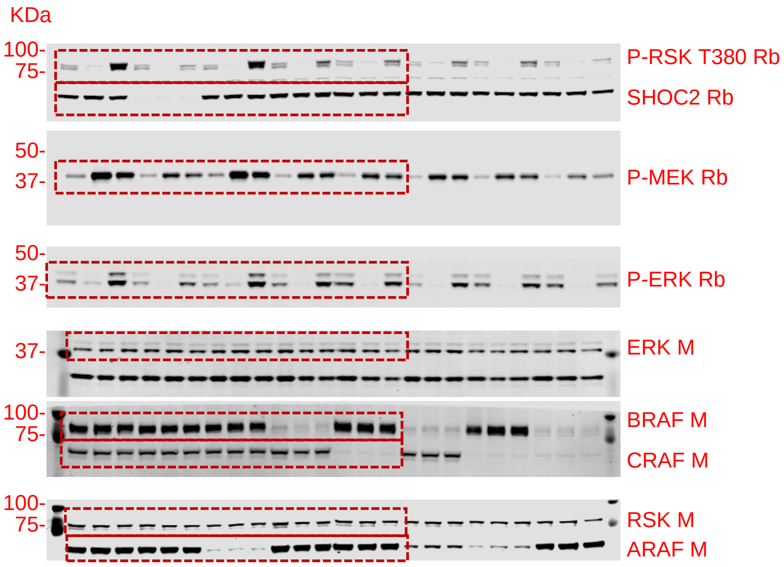

Supplementary Figure 14 | Uncropped western blots of the most important blots Fig. 5e
