# Supplementary material for: SHOC2 phosphatase-dependent RAF dimerization mediates resistance to MEK inhibition in RAS-mutant cancers
Source: Nat Commun. 2019 Jun 10;10:2532. doi: 10.1038/s41467-019-10367-x (PMC6557854; doi:10.1038/s41467-019-10367-x)
Supplement: Supplementary file 2 — Reporting Summary [file 41467_2019_10367_MOESM2_ESM.pdf]

nature research

Corresponding author(s): Pablo Rodriguez-Viciana



# Reporting Summary

Nature Research wishes to improve the reproducibility of the work that we publish. This form provides structure for consistency and transparency in reporting. For further information on Nature Research policies, see Authors & Referees and the Editorial Policy Checklist.

## Statistical parameters

When statistical analyses are reported, confirm that the following items are present in the relevant location (e.g. figure legend, table legend, main text, or Methods section).

| n/a | Confirmed | |
|---|---|---|
| ☐ | ☒ | The exact sample size ($n$) for each experimental group/condition, given as a discrete number and unit of measurement |
| ☐ | ☒ | An indication of whether measurements were taken from distinct samples or whether the same sample was measured repeatedly |
| ☐ | ☒ | The statistical test(s) used AND whether they are one- or two-sided<br>*Only common tests should be described solely by name; describe more complex techniques in the Methods section.* |
| ☒ | ☐ | A description of all covariates tested |
| ☒ | ☐ | A description of any assumptions or corrections, such as tests of normality and adjustment for multiple comparisons |
| ☐ | ☒ | A full description of the statistics including central tendency (e.g. means) or other basic estimates (e.g. regression coefficient) AND variation (e.g. standard deviation) or associated estimates of uncertainty (e.g. confidence intervals) |
| ☒ | ☐ | For null hypothesis testing, the test statistic (e.g. $F$, $t$, $r$) with confidence intervals, effect sizes, degrees of freedom and $P$ value noted<br>*Give P values as exact values whenever suitable.* |
| ☒ | ☐ | For Bayesian analysis, information on the choice of priors and Markov chain Monte Carlo settings |
| ☒ | ☐ | For hierarchical and complex designs, identification of the appropriate level for tests and full reporting of outcomes |
| ☒ | ☐ | Estimates of effect sizes (e.g. Cohen's $d$, Pearson's $r$), indicating how they were calculated |
| ☐ | ☒ | Clearly defined error bars<br>*State explicitly what error bars represent (e.g. SD, SE, CI)* |

*Our web collection on statistics for biologists may be useful.*

## Software and code

Policy information about availability of computer code

| | |
|---|---|
| Data collection | No Software was used in this study |
| Data analysis | No Software was used in this study |

For manuscripts utilizing custom algorithms or software that are central to the research but not yet described in published literature, software must be made available to editors/reviewers upon request. We strongly encourage code deposition in a community repository (e.g. GitHub). See the Nature Research guidelines for submitting code & software for further information.

## Data

Policy information about availability of data

All manuscripts must include a data availability statement. This statement should provide the following information, where applicable:

- Accession codes, unique identifiers, or web links for publicly available datasets
- A list of figures that have associated raw data
- A description of any restrictions on data availability

The authors declare that [the/all other] data supporting the findings of this study are available within the paper [and its supplementary information files]. For more information please contact the corresponding author.

# Field-specific reporting

Please select the best fit for your research. If you are not sure, read the appropriate sections before making your selection.

☒ Life sciences ☐ Behavioural & social sciences ☐ Ecological, evolutionary & environmental sciences

For a reference copy of the document with all sections, see nature.com/authors/policies/ReportingSummary-flat.pdf

# Life sciences study design

All studies must disclose on these points even when the disclosure is negative.

| | |
|---|---|
| Sample size | No sample size calculations were performed because this was not required for this study. |
| Data exclusions | No data exclusions. |
| Replication | Replicate experiments using multiple approaches were used and are described for the findings of this study. All replicates are reported. |
| Randomization | Samples/ Organisms were randomly assigned to experimental groups/ cohorts for all experiments in this study. |
| Blinding | Blinding was not required for this study. |

# Reporting for specific materials, systems and methods

## Materials & experimental systems

| n/a | Involved in the study | |
|---|---|---|
| ☒ | ☐ | Unique biological materials |
| ☐ | ☒ | Antibodies |
| ☐ | ☒ | Eukaryotic cell lines |
| ☒ | ☐ | Palaeontology |
| ☐ | ☒ | Animals and other organisms |
| ☒ | ☐ | Human research participants |

## Methods

| n/a | Involved in the study | |
|---|---|---|
| ☒ | ☐ | ChIP-seq |
| ☐ | ☒ | Flow cytometry |
| ☒ | ☐ | MRI-based neuroimaging |

## Antibodies

Antibodies used

AKT (pan) Cell Signaling Technology 2920 Mouse
AKT P-S473 Cell Signaling Technology 4060 Rabbit
ARAF Santa Cruz sc-166771 Mouse
ARAF Santa Cruz sc-408 Rabbit
β-Actin Santa Cruz sc-47778 Mouse
BAD S112 Cell Signaling Technology 9291 Rabbit
BIM Cell Signaling Technology 2933 Rabbit
BRAF Santa Cruz sc-5284 Mouse
BRAF Santa Cruz sc-9002 Rabbit
BRAF P-T753 Abcam ab138399 Rabbit
CRAF Santa Cruz sc-7267 Mouse
CRAF BD Biosciences 610152 Mouse
CRAF Santa Cruz sc-133 Rabbit
CRAF P-S289/296/301 Cell Signaling Technology 9431 Rabbit
EGFR Santa Cruz sc-373746 Mouse
EGFR P-T669 Cell Signaling Technology 3056 & 8808 Rabbit
ERK ½ Cell Signaling Technology 9102 Rabbit
ERK ½ Cell Signaling Technology 9107 Mouse
ERK 1/2 P-T202/Y204 Cell Signaling Technology 9101 Rabbit
FLAG Sigma F1365 Mouse
GAPDH Genetex GT239 Mouse
KRAS Santa Cruz sc-30 Mouse
MEK1 Santa Cruz sc-6250 Mouse
MEK2 Santa Cruz sc-13159 Mouse
MEK ½ Cell Signaling Technology 4694 Rabbit

MEK 1/2 P-S217/221 Cell Signaling Technology 9121 & 9154 Rabbit
MYC-TAG Cell Signaling Technology 9B11 Mouse
PARP BD Biosciences 556494 Mouse
PARP (cleaved) Cell Signaling Technology 9541 Rabbit
RSK1 Santa Cruz sc-231 Rabbit
RSK2 Santa Cruz sc-9986 Mouse
RSK1 P-S380 Cell Signaling Technology 11989 Rabbit
YB1 Santa Cruz sc-398340 Mouse
YB1 P-S102 Cell Signaling Technology 2900 Rabbit

Validation

All commercially available antibodies listed above are validated for use by Western blot by the manufacturer. In house antibodies including BRAF P-S365 were generated by immunisation of rabbits with a phosphpo-peptide corresponding to the appropriate region of BRAF (Epitomics/Abcam). SHOC2 antibody was generated as described previously (Rodriguez-Viciana, P et al 2006).

## Eukaryotic cell lines

Policy information about cell lines

Cell line source(s)

Cell services Francis Crick Institute

Authentication

STR profiling was used for all human lines described in this study by Francis Crick cell services. STR profiling is then cross-referenced against any available published profiles. In addition cell services use a species identification test for all lines to confirm the correct species and to ensure that there isn't inter-species cross-contamination.
As part of the cross-reference process cell services also double check that the lines are not listed on any 'mis-identified cell lists' namely ICLAC (International Cell Line Authentication Committee) website.

Mycoplasma contamination

All human cell lines tested negative for Mycoplasma by Francis Crick cell services

Commonly misidentified lines
(See ICLAC register)

*Name any commonly misidentified cell lines used in the study and provide a rationale for their use.*

## Animals and other organisms

Policy information about studies involving animals; ARRIVE guidelines recommended for reporting animal research

Laboratory animals

C57BL/6 Male and Female 6-12 weeks of age (CharlesRiver) For AdenoCre Lung tumor experiments
Athymic Nude , Female, 6-weeks of age (CharlesRiver) For subcutaneous xenograft assays
Fox Chase SCID Beige, Female, (CharlesRiver) For lung colonization assays following tail-vein injections

Wild animals

This study did not involve wild animals

Field-collected samples

This study did not involve samples collected from the field

## Flow Cytometry

### Plots

Confirm that:

☒ The axis labels state the marker and fluorochrome used (e.g. CD4-FITC).

☒ The axis scales are clearly visible. Include numbers along axes only for bottom left plot of group (a 'group' is an analysis of identical markers).

☒ All plots are contour plots with outliers or pseudocolor plots.

☒ A numerical value for number of cells or percentage (with statistics) is provided.

### Methodology

Sample preparation

Adherent human cells were trypsinized, gently washed and stained with Annexin V-FITC and PI in Annexin V binding buffer (approximatly 1*10^6 cells per 500ul buffer) for 10' at 4C in the dark.

Instrument

BD LSR Fortessa X 20

Software

FloJoLLC Flow Jo 10.4.2

Cell population abundance

Abundance of relevant cell populations is provided within the figure as a (%) of the total cell input.

Gating strategy

Forward scatter (FSC) and side scatter (SSC) gates were used to identify a single cell population. PI and AnnexinV-FITC allowed sorting of live/ apoptotic and dead cell populations. Gating strategy to be added to supplementary data.

☒ Tick this box to confirm that a figure exemplifying the gating strategy is provided in the Supplementary Information.
